# Root coverage using a connective tissue graft with epithelial striation in combination with enamel matrix derivatives - a long-term retrospective clinical interventional study

**DOI:** 10.1186/s12903-019-0849-7

**Published:** 2019-07-15

**Authors:** Knut Adam, Ingmar Staufenbiel, Werner Geurtsen, Hüsamettin Günay

**Affiliations:** Department of Conservative Dentistry, Periodontology and Preventive Dentistry, Hannover Medical School, Carl-Neuberg-Str. 1, 30625 Hannover, Germany

**Keywords:** Connective tissue graft with epithelial striation, Coronally advanced flap, Enamel matrix derivatives, Plastic periodontal surgery, Long-term stability

## Abstract

**Background:**

The application of a connective tissue graft with epithelial striation (CTG-ES) has been shown to improve the outcome of root coverage (RC) using the coronally advanced flap (CAF) and adjunctive administration of enamel matrix derivatives (EMD). Aim of the present study was to evaluate the long-term (mean: 16.19 ± 1.80 years, range: 13 to 18 years) stability of this treatment method with special focus on the location of the gingival margin and the width of keratinized tissue (WKT).

**Methods:**

16 patients (10 female, 6 male, aged 35.36 ± 14.70 years at surgery) with 25 Miller class I or II gingival recession (GR) defects were treated using the CAF combined with the CTG-ES and EMD. The clinical measurements recorded at baseline (t0), 6 months (t1), and 13 to 18 years (t2) after surgery included recession depth (RED), probing pocket depth (PPD), clinical attachment level (CAL), and WKT. In addition, the number of sites with complete RC (CRC) and the mean RC (MRC) were documented at t1 and t2. The statistical analysis was performed using a linear mixed model.

**Results:**

The RED (t0: 4.52 ± 1.56 mm; t1: 0.36 ± 0.76 mm; t2: 0.30 ± 0.60 mm) and CAL (t0: 6.16 ± 1.62 mm; t1: 1.86 ± 0.87 mm; t2: 1.54 ± 0.92 mm) were significantly reduced at t1 and t2 compared to t0 (*p* <  0.001). The PPD was significantly reduced at t2 compared to t0 (*p* = 0.016). The WKT (t0: 1.18 ± 1.28 mm; t1: 3.26 ± 0.98 mm; t2: 4.26 ± 1.83 mm) significantly increased from t0 to t1, from t0 to t2 (p <  0.001) and from t1 to t2 (*p* = 0.007). A CRC was recorded at 19 sites (76.0%) at t1 and t2. The MRC was 93.6 ± 12.8% at t1 and 93.3 ± 13.3% at t2.

**Conclusions:**

The use of the CAF combined with CTG-ES and EMD leads to stable long-term outcomes on teeth with Miller Class I or II GR defects. The CTG-ES represents a hybrid graft with increased position stability and advantageous properties for the healing process. We assume that the ES is responsible for the increase of the WKT.

## Background

Gingival recessions (GR) developing at the buccal site of teeth are frequently observed in patients with a high standard of oral hygiene [1–3]. The aetiology of GR is multifactorial and includes anatomical, physiological, and pathological factors [4]. Patients with a thin periodontal phenotype frequently show a dehiscence of the alveolar bone, deficient soft tissue volume, and lack of keratinized tissue (KT) at the buccal site of teeth [4, 5]. When these predisposing anatomical factors coincide with causal pathological factors, like traumatic tooth brushing, intra- or perioral piercings, bad habits, malocclusion or inflammation due to increased plaque accumulation, GR may develop [4]. The treatment of GR defects is necessary to help patients suffering from the aesthetic impairment, dentin hypersensitivity or both and to avoid potential sequelae, like gingivitis, periodontitis, root caries, and abrasion defects. A satisfying ‘red-white-aesthetic’ and a decreased sensitivity to thermal and chemical stimuli can be achieved by root coverage (RC) procedures [6, 7]. There is a controversy in the scientific literature about how much KT is required to maintain periodontal health and prevent the development of GR. It has been shown that sites without attached gingiva do not develop GR, when an optimal and atraumatic plaque control is maintained [8]. However, when GR are present and RC procedures are performed at sites with a deficient attached gingiva, soft tissue autografts are regularly used to increase the soft tissue volume and the width of KT (WKT) [9]. Free gingival grafts (FGG), which have been applied for decades, predictably lead to KT. But they are associated with aesthetic limitations. Thus, FGG harvested from the lateral palate significantly differ from the adjacent resident soft tissues concerning colour and texture and have a ‘patch-like’ appearance [5]. Sub-epithelial connective tissue grafts (CTG) in combination with a coronally advanced flap (CAF) may result in excellent aesthetic outcomes, but seem to be less effective in increasing the WKT [10, 11].

Our group introduced the CTG with an epithelial striation (CTG-ES), which combines the FGG and the CTG in one single hybrid graft [12]. We routinely use it in combination with the CAF and enamel matrix derivatives (EMD) for the RC of GR on teeth with a deficient soft tissue volume and a lack of KT. In contrast to the CTG, the CTG-ES is only partially covered by the CAF and the ES segment is located coronally to the flap margin.

Over time, the recurrence of GR is a frequently observed phenomenon after RC using the CAF [13]. Accordingly, Pini-Prato et al. [14] observed in a long-term study that GR increased in 53% of treated sites from 6 months to 8 years after surgery. They reported that the apical shift of the gingival margin was accompanied by a decrease of KT. The long-term stability of RC using the CAF combined with the CTG-ES and EMD in patients with Miller class I or II GR defects has not been investigated yet. Therefore, hypothesis of the present retrospective clinical interventional study was that this treatment method leads to a stable position of the gingival margin and a consistent amount of KT from 6 months to more than 12 years after surgery.

## Methods

### Study participants

In 16 patients (10 women and 6 men) with a mean age of 35.36 ± 14.70 years at surgery (range: 16 to 66 years), RC was conducted on teeth with Miller class I or II GR defects using the CAF with adjunctive application of EMD. In all cases of this clinical interventional study, a CTG-ES was used to increase the soft tissue volume and the WKT. All surgical procedures were performed by one single experienced periodontist (HG).

As the success of mucogingival surgery is substantially dependant on patient’s compliance, a meticulous presurgical selection was conducted according to the following exclusion criteria: (1) smoking, (2) systemic diseases with a known impact on the periodontal tissues (e.g. diabetes mellitus), and (3) poor oral hygiene. For the retrospective analysis, a follow-up period of at least 12 years was demanded.

### Clinical parameters

The clinical measurements were rounded to the nearest half of a millimetre and included recession depth (RED), probing pocket depth (PPD), clinical attachment level (CAL), and width of keratinized tissue (WKT) recorded at the mid-buccal site of the affected tooth. Schiller’s iodine solution was used to stain the alveolar mucosa, highlight the mucogingival junction (MGJ), and facilitate the measurement of the WKT. A complete RC (CRC) was documented when the cemento-enamel-junction (CEJ) was not exposed. The mean RC (MRC) was calculated using the formula: MRC = [RED(t0) – RED(t1 or t2)]/RED(t0) × 100 [%]. The clinical parameters were recorded before surgery (t0) as well as 6 months (t1) and 13 to 18 years (t2) after surgery using a WHO periodontal probe (GY12, Deppeler, Rolle, Switzerland).

### Surgical procedure

**Fig. 1:**
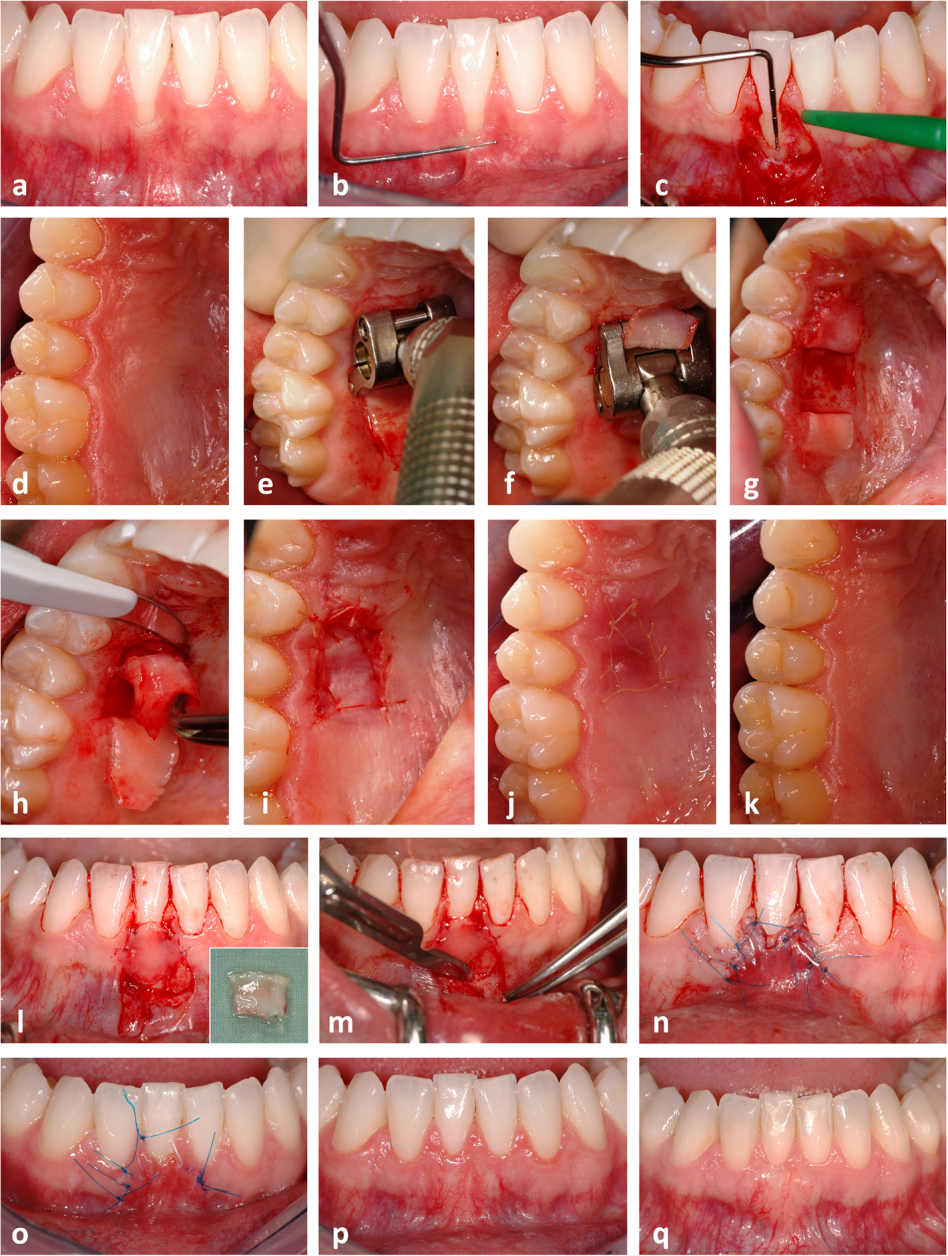
Gingival recession (GR) at the lower right first incisor (**a**). Note the lack of keratinized tissue illustrated by a periodontal probe and the rolling technique (**b**) and the bone dehiscence after preparation and mobilisation of the trapezoidal flap (**c**). The connective tissue graft with epithelial striation (CTG-ES) was harvested at the donor site using a mucotome and the modified trapdoor technique (**d**-**k**). The preparation of the epithelial pedicle flap was conducted with a mucotome blade with a width of 6 mm in anterior-posterior direction (**e**). The preparation of the CTG-ES was performed with a mucotome blade with a width of 8 mm in posterior-anterior direction (**f**). Note the ES adjacent to the marginal gingiva (**h**). CTG-ES fixed at the recipient site with resorbable sutures covering the bone dehiscence and the GR defect (**l**). Note the even and uniform thickness of the graft and the epithelial striation (ES) of about 2 mm width (l, window). Periosteal slitting at the mucogingival junction (**m**). Recipient site at the end of surgery (**n**) as well as 8 days (**o**), 6 months (**p**), and 13 years (**q**) after surgery

**Fig. 2:**
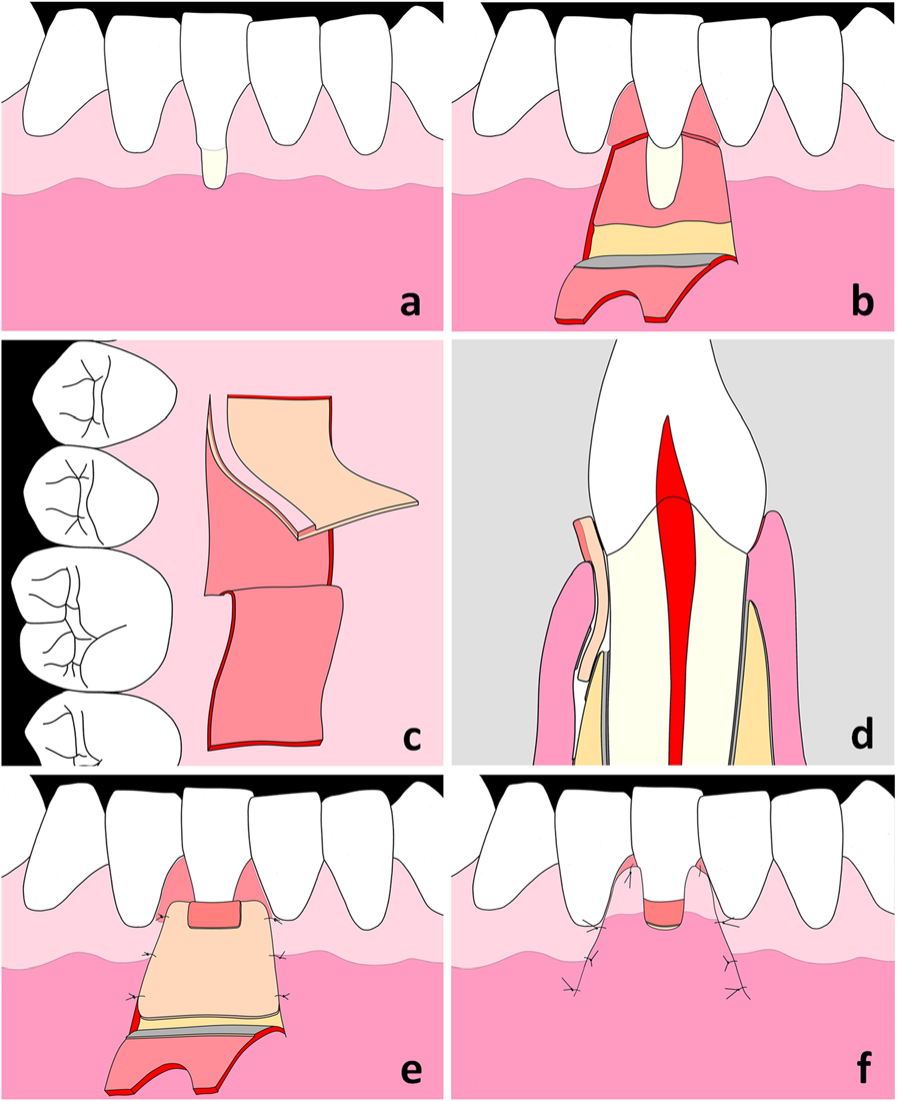
Schematic illustration of the surgical technique: Gingival recession (GR) at the lower right first incisor (**a**). At the recipient site, the trapezoidal flap is initially dissected as mucosal flap and 2 mm apically to the bone crest as mucoperiosteal flap. Note the de-epithelialization of the anatomical papillae (**b**). The connective tissue graft with epithelial striation (CTG-ES) is harvested at the donor site in two steps. Firstly, the epithelial flap is elevated in anterior-posterior direction using a mucotome blade with a width of 6 mm. Secondly, the CTG-ES is dissected in posterior-anterior direction using a mucotome blade with a width of 8 mm (**c**). The ideal positioning of the CTG-ES in a cross sectional view: The most coronal part of the CTG-ES projects above the cemento-enamel junction (CEJ) by 2 mm. Note that only the CTG segment of the graft is covered by the trapezoidal flap (**d**). The CTG-ES is positioned at the recipient bed and fixed with resorbable sutures. Note the partial de-epithelialization of the ES mesially and distally to the GR defect (**e**). Situation at the end of the surgical procedure: The flap is coronally advanced and fixed with sling sutures at the papillae and interrupted sutures at the releasing incisions (**f**)

The pre-surgical treatment consisted of a full-mouth professional dental cleaning and oral hygiene instructions, which aimed at eliminating the etiologic habits. Patients rinsed for 1 min with a 0.2% chlorhexidine digluconate solution immediately before surgery. After local anaesthesia at the donor and recipient sites (Xylocaine® 2% DENTAL with adrenaline 1:100000, DENTSPLY De Trey GmbH, Konstanz, Germany), mechanical debridement with hand instruments (scalers and curettes) and an air-scaler (SONICflex, KaVo, Biberach, Germany) was performed at the exposed root surface(s). An intrasulcular incision was carried out on the buccal aspect of the affected tooth and extended horizontally to the basis of the mesial and distal papilla in order to surgically create new papillae. Afterwards, two oblique releasing incisions, which started at the end of the horizontal incisions and reached beyond the MGJ, were conducted. The resulting trapezoidal flap was mobilized – initially as mucosal flap and 2 mm apically from the bone crest as mucoperiosteal flap – using a microsurgical blade and raspatorium. A periosteal slitting was carried out in mesio-distal direction to dissect residual muscle fibres and to allow a passive, tension-free displacement of the flap. At the donor site reaching from the distal aspect of the first upper bicuspid to the mesial aspect of the first upper molar, a CTG-ES was harvested from the palatal masticatory mucosa using the modified trapdoor technique (MTT) [12]. Mucotome blades of different widths were used in a two-step-approach to create the CTG-ES. In the first step, a mucotome blade with a width of 6 mm was used to elevate an epithelial flap. In the second step, a mucotome blade with a width of 8 mm was used at the same position as before, so that a CTG with an ES of ideally 2 mm width was created. Afterwards, the CTG-ES was harvested from the donor site with a precise cut and the epithelial flap was repositioned and secured with a continuous suture. Individual modifications of the CTG-ES were conducted on a sterile glass plate under continuous humidification with sterile physiologic saline solution. Glandular and adipose tissues were removed. In addition, the ES was partially de-epithelialized so that an overlap between CAF and ES was reduced to a minimal extent. As soon as the CTG-ES had the desired size and shape, the regenerative procedure consisting of the application of 24% ethylene-diamine-tetra-acetic (EDTA) acid gel (PrefGel, Straumann, Freiburg, Germany) and EMD (Emdogain, Straumann, Freiburg, Germany) was applied at the GR defect. Afterwards, the mesial and distal anatomical papillae were de-epithelialized with a scalpel. The CTG-ES was placed at the recipient site covering the CEJ by 1 to 2 mm and fixed with resorbable sutures (Vicryl Rapide 7–0, Ethicon, Norderstedt, Germany). Finally, the flap was coronally advanced and fixed with interrupted sutures at the releasing incisions and sling sutures at the papillae (Prolene 7–0, Ethicon, Norderstedt, Germany; GORE-TEX Suture CV-6, W. L. Gore & Associates, Putzbrunn, Germany). It is important to note that the ES was located coronally to the flap margin and that the flap should overlap the ES as little as possible. In case of adjacent GR defects, the CTG-ES was extended and modified according to the requirements of the individual situation. A sterile gauze swab soaked in sterile physiologic saline solution was used to compress the operation area for 1 min, in order to reduce the formation of a hematoma and promote the healing process. No periodontal dressing was applied. The individual steps of the surgical procedure are shown in a representative clinical case (Fig. 1) and corresponding schematic illustrations (Fig. 2).

### Postsurgical care

The patients were instructed to rinse for 1 min with an isotonic sodium chloride solution (NaCl 0.9%, Braun, Melsungen, Germany) at regular intervals during the first day. Subsequently, irrigation for 1 min with a 0.2% chlorhexidine digluconate solution was recommended twice daily. Patients were advised that the solutions were to be in contact with the surgical area during this time, but physical stresses caused by excessive movements of the mouth should be avoided. Mechanical plaque control (tooth-brushing, flossing etc.) and trauma of any kind were prohibited at the surgical site for at least three weeks. Afterwards, the patients were allowed to resume mechanical plaque control using a very soft toothbrush. The sutures were removed 8 to 14 days after surgery. All patients were enrolled in a systematic, risk-oriented recall program with a regular full-mouth prophylaxis.

### Statistical analysis

The recorded data were documented and analysed using the IBM SPSS Statistics 25 software (IBM, Ehningen, Germany). Descriptive data are expressed as mean values ± standard deviation. The statistical analysis was performed using a linear mixed model, in which the patients and teeth were included with random effects. The model specification considers teeth as nested within subjects (patients). Time was applied as a repeated factor. In addition, the factor ‘gender’ and the covariate ‘age at surgery’ were included in the model. The measurements at repeated time points were compared by applying a Bonferroni adjustment. A value of *p* ≤ 0.05 was considered statistically significant.

## Results

A total of 25 GR defects were included in the study; 11 were located in the upper jaw (2 incisors, 9 canines) and 14 in the lower jaw (6 incisors, 4 canines, 4 bicuspids). The linear mixed model revealed that the factor ‘gender’ and the covariate ‘age at surgery’ did not significantly influence the changes of the clinical parameters. The RED was significantly reduced at t1 [ΔRED(t1-t0) = − 4.16 mm; *p* <  0.001] and t2 [ΔRED(t2-t0) = − 4.22 mm; *p* <  0.001] compared to t0. The PPD did not significantly change between t0 and t1 [ΔPPD(t1-t0) = − 0.14 mm; *p* = 0.435], but was significantly decreased at t2 compared to t0 [ΔPPD(t2-t0) = − 0.40 mm; *p* = 0.016]. A significant CAL gain was recorded at t1 [ΔCAL(t1-t0) = − 4.30 mm; *p* <  0.001] and t2 [ΔCAL(t2-t0) = − 4.62 mm; *p* <  0.001] compared to t0. The WKT was significantly increased at t1 [ΔWKT(t1-t0) = 2.08 mm; *p* <  0.001] and t2 [ΔWKT(t2-t0) = 3.08 mm; *p* <  0.001] compared to t0. In addition, the WKT significantly increased from t1 to t2 [ΔWKT(t2-t1) = 1.00 mm; *p* = 0.007]. A CRC was documented in 19 out of 25 sites (76.0%) at t1 and t2. The MRC was 93.6 ± 12.8% at t1 and 93.3 ± 13.3% at t2. The data of the clinical parameters are presented in Table 1.

**Table 1:** Clinical parameters recorded at baseline (t0), 6 months (t1), and 13 to 18 years (t2) after surgery. (PPD: probing pocket depth; RED: recession depth; CAL: clinical attachment level; WKT: width of keratinized tissue; MV: mean value; SD: standard deviation); *: linear mixed model

		times of the assessment [MV ± SD]			Significance level / *p*-value			
	N	t0	t1	t2	t0 vs. t1 vs. t2*	t0 vs. t1*	t0 vs. t2*	t1 vs. t2*
PPD	25	1.64 ± 0.45	1.50 ± 0.46	1.24 ± 0.61	0.020	0.435	0.016	0.108
RED	25	4.52 ± 1.56	0.36 ± 0.76	0.30 ± 0.60	< 0.001	< 0.001	< 0.001	0.842
CAL	25	6.16 ± 1.62	1.86 ± 0.87	1.54 ± 0.92	< 0.001	< 0.001	< 0.001	0.245
WKT	25	1.18 ± 1.28	3.26 ± 0.98	4.26 ± 1.83	< 0.001	< 0.001	< 0.001	0.007

## Discussion

The RC and gingival augmentation performed in the present study led to a stable position of the gingival margin and an increase of KT more than 12 years after surgery. Therefore, the hypothesis of our study was confirmed. Patients with a thin periodontal phenotype and a narrow band of KT are prone to the development of GR [15]. At teeth affected by GR, gingival augmentation procedures aim at preventing a progression of the defect by increasing the soft tissue volume and the WKT. The additional use of a CTG during RC with the CAF was shown to induce an increase of KT [16]. However, histological examinations in humans revealed that a CTG does not lead to keratinization of the overlying alveolar mucosa [11]. This histological observation is in accordance with our clinical experience. When patients were treated with the CAF, CTG and EMD, we observed an increase of the soft tissue volume, but not an increase of the KT in the area, where the CTG was positioned. FGG can be used to increase the WKT predictably. However, a FGG is frequently associated with an unsatisfactory colour and texture match at the recipient site and postoperative problems at the donor site [17]. Therefore, it is less accepted than the CTG. The CTG-ES used in the present study combines the advantages of both, the FGG and the CTG, in one single hybrid graft. In contrast to the FGG, harvesting of the CTG-ES does not cause an open wound surface at the donor site, which requires healing through granulation. The denuded sub-epithelial connective tissue is covered by the epithelial flap and, thus, patients generally do not suffer from considerable postoperative pain at the donor site.

The data of our study provide strong evidence that the use of the CAF combined with a CTG-ES and EMD leads to stable long-term results on teeth with Miller Class I or II GR defects. Thus, the CAL significantly improved throughout the entire observation period (CAL gain: 4.30 mm from t0 to t1; 0.32 mm from t1 to t2). A detailed look at the data revealed that the significant CAL changes were mainly produced by the RED reduction and to a little extent by the PPD reduction. Cheng et al. [16] published a meta-analysis that investigated the RC of Miller class I, II and III GR defects using the CAF in combination with a CTG and/or EMD. They noticed that a higher PPD reduction tend to occur when EMD were additionally used. This observation is supported by the results of our study. Moreover, a CRC was obtained in 19 (76.0%) out of our 25 treated sites and a MRC of 93.3% was found 13 to 18 years after surgery. Other groups investigated the combination CAF, CTG and EMD for the treatment of GR and described a lower percentage of CRC (ranging from 56.5 to 70%) 1 year after surgery [18–20]. The higher percentage of CRC in our study may be explained by the use of the CTG-ES. When the CTG-ES was harvested at the donor site using the mucotome in a two-step-approach, a hybrid graft with two segments was created: one segment with and one segment without an epithelial layer. Thereby, a remarkable edge was generated at the boundary of these segments. We hypothesize that this edge provided additional position stability to the graft and promoted the subsequent regenerative healing process. All grafts showed an even, uniform thickness of about 1 to 1.5 mm due to the harvesting technique using a mucotome. We assume that this homogeneous graft design facilitated the initial nourishment of the graft via diffusion, favoured the revascularization from the recipient bed, and allowed an aesthetically appealing integration of the graft within the early healing and subsequent maturation process.

There are only a few studies addressing the long-term stability of the surgical treatment of GR. Most of these studies examined the application of a FGG [21, 22]. In a recently published article, 21 class I and 24 class III GR defects were treated in the maxilla using the CAF plus CTG and re-evaluated 20 years after surgery [23]. In the group of class I GR, a CRC was achieved in 12 sites (57.14%) at the 1-year follow-up and in 10 sites (47.62%) at the 20-year follow-up; the MRC decreased from 82.37% (1-year follow-up) to 77.62% (20-year follow-up). In the group of class III GR, a CRC was observed in 5 teeth (20.83%) 1 year as well as 20 years after surgery; the MRC decreased from 66.55% (1-year follow-up) to 58.18% (20-year follow-up). The authors conducted a logistic regression analysis and observed that the recurrence of GR was associated with non-carious cervical lesions, smoking, and sites with a WKT < 2 mm. The last finding suggests again that an adequate amount of KT is pivotal to prevent a recurrence of GR and to allow a long-term stability of the treatment outcome.

**Fig. 3:**
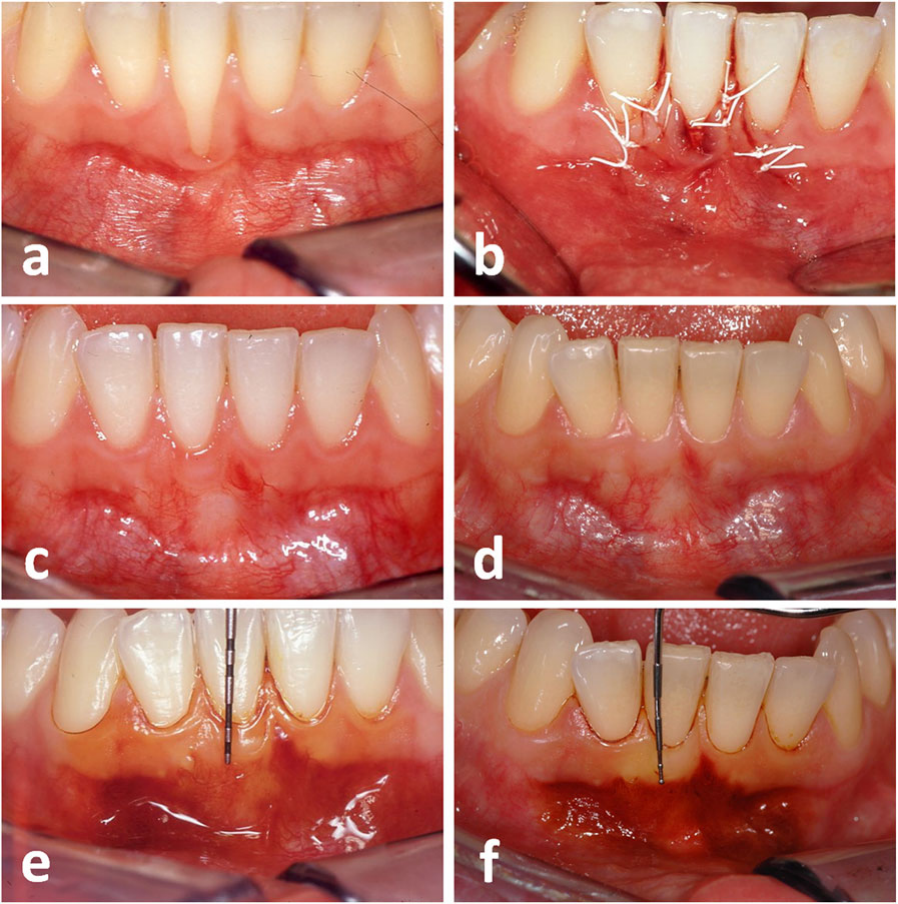
Gingival recession (GR) at the lower right first incisor (**a**). Recipient site at the end of surgery (**b**), 6 months (**c**), and 18 years (**d**) after surgery. Staining of the alveolar mucosa using Schiller’s iodine solution 6 months (**e**) and 18 years (**f**) after surgery. Note the harmonious symmetric alignment of the mucogingival junction and the increase of keratinized tissue

In the present study, the CTG-ES sustainably increased the WKT, presumably through the ES segment and the soft tissue volume through the CTG segment. As the WKT significantly increased throughout the entire observation period (WKT gain: 2.08 mm from t0 to t1; 1.00 mm from t1 to t2), it seems likely that the CTG-ES has a long-lasting effect on the formation of KT. There are two possible mechanisms that can increase the WKT during the long-term maturation process. We believe that the increase of KT is mainly caused by the return of the MGJ to its original position as hypothesized by Zucchelli and De Sanctis [24]. An example for this phenomenon can be seen in Fig. 3, where the band of KT is aligned in an axisymmetric way. Creeping attachment, which is defined as a coronal shift of the gingival margin, is the second possible mechanism. Since there were sites, where the RED decreased and the WKT simultaneously increased to a greater extent between t1 and t2, it can be concluded that both mechanisms have contributed to the increase of KT in such cases.

To the best of our knowledge, there is only one further study that assessed a hybrid graft for the treatment of GR. Cortellini et al. [5] used a partly epithelialized free gingival graft (PE-FGG) for the treatment of 12 single GR (12 patients) and 16 multiple GR (7 patients) at lower incisors. They observed in both groups a significant RED decrease and a significant WKT increase 1 year after surgery. The MRC accounted for 94% in the group of single GR and 96% in the group of multiple GR. Furthermore, they documented that the PE-FGG resulted in good aesthetic outcomes, which were comparable to that of CAF approaches. There are considerable differences between both methods (PE-FGG vs. CTG-ES) with regard to the harvesting of the grafts and the preparation of the recipient sites. However, the results of both methods suggest that the use of a hybrid graft with an epithelial component has a stimulating effect on the formation of KT and does not negatively influence the aesthetic outcome. Comparative studies that could unequivocally prove or disprove the superiority of a hybrid graft are missing so far. Well-designed prospective, randomized, controlled, clinical trials that compare the long-term efficacy of both graft designs (CTG vs. CTG-ES) in terms of CRC, MRC, and WKT are needed to clarify this uncertainty.

A further explanation for the stability of the treatment outcome could be that all patients of the present study participated in a systematic recall program in our department or in private practices. The supportive care was generally conducted in 3- to 6-month-intervals and consisted of a professional full-mouth dental cleaning and individual oral hygiene instructions. Accordingly, other studies have demonstrated that the compliance with a supportive care program is crucial for the maintenance of stable RC outcomes [21, 24].

## Conclusions

RC with the CAF in combination with a CTG-ES and EMD sustainably improves the mucogingival conditions of teeth affected by GR. Thus, RED, PPD, and CAL were significantly reduced 13 to 18 years after surgery. In addition, a significant layer of KT was achieved. We assume that this was due to the stimulating effect of the ES.

## Data Availability

The datasets generated and/or analysed during the current study are not publicly available but are available from the corresponding author on reasonable request.

## Abbreviations

CAF: coronally advanced flap
CAL: clinical attachment level
CEJ: cemento-enamel-junction
CRC: complete root coverage
CTG: connective tissue graft
CTG-ES: connective tissue graft with epithelial striation
EMD: enamel matrix derivatives
GR: gingival recession
KT: keratinized tissue
MGJ: mucogingival junction
MRC: mean root coverage
MTT: modified trapdoor technique
PE-FGG: partly epithelialized free gingival graft
PPD: probing pocket depth
RC: root coverage
RED: recession depth
WKT: width of keratinized tissue

## References

[1] Serino G, Wennström JL, Lindhe J, Eneroth L (1994). The prevalence and distribution of gingival recession in subjects with a high standard of oral hygiene. J Clin Periodontol.

[2] Albandar JM, Kingman A (1999). Gingival recession, gingival bleeding, and dental calculus in adults 30 years of age and older in the United States, 1988-1994. J Periodontol.

[3] Kassab MM, Cohen RE (2003). The etiology and prevalence of gingival recession. J Am Dent Assoc.

[4] Zucchelli Giovanni, Mounssif Ilham (2015). Periodontal plastic surgery. Periodontology 2000.

[5] Cortellini P, Tonetti M, Prato GP (2012). The partly epithelialized free gingival graft (pe-fgg) at lower incisors. A pilot study with implications for alignment of the mucogingival junction. J Clin Periodontol.

[6] Chambrone L, Pannuti CM, Tu YK, Chambrone LA (2012). Evidence-based periodontal plastic surgery. II. An individual data meta-analysis for evaluating factors in achieving complete root coverage. J Periodontol.

[7] Douglas de Oliveira DW, Oliveira-Ferreira F, Flecha OD, Gonçalves PF (2013). Is surgical root coverage effective for the treatment of cervical dentin hypersensitivity? A systematic review. J Periodontol.

[8] Wennström J, Lindhe J (1983). Role of attached gingiva for maintenance of periodontal health. Healing following excisional and grafting procedures in dogs. J Clin Periodontol.

[9] Zuhr O, Bäumer D, Hürzeler M (2014). The addition of soft tissue replacement grafts in plastic periodontal and implant surgery: critical elements in design and execution. J Clin Periodontol.

[10] Stefanini M, Zucchelli G, Marzadori M, de Sanctis M (2018). Coronally advanced flap with site-specific application of connective tissue graft for the treatment of multiple adjacent gingival recessions: a 3-year follow-up case series. Int J Periodontics Restorative Dent.

[11] Cummings LC, Kaldahl WB, Allen EP (2005). Histologic evaluation of autogenous connective tissue and acellular dermal matrix grafts in humans. J Periodontol.

[12] Günay H, Dogan S, Geurtsen W (2008). Harvesting technique using a mucotome and modified surgical procedure for root coverage with enamel matrix derivatives with and without a connective tissue graft. Int J Periodontics Restorative Dent.

[13] Leknes KN, Amarante ES, Price DE, Bøe OE, Skavland RJ, Lie T (2005). Coronally positioned flap procedures with or without a biodegradable membrane in the treatment of human gingival recession. A 6-year follow-up study. J Clin Periodontol.

[14] Pini-Prato G, Franceschi D, Rotundo R, Cairo F, Cortellini P, Nieri M (2012). Long-term 8-year outcomes of coronally advanced flap for root coverage. J Periodontol.

[15] Fischer KR, Künzlberger A, Donos N, Fickl S, Friedmann A (2018). Gingival biotype revisited-novel classification and assessment tool. Clin Oral Investig.

[16] Cheng GL, Fu E, Tu YK, Shen EC, Chiu HC, Huang RY, Yuh DY, Chiang CY (2015). Root coverage by coronally advanced flap with connective tissue graft and/or enamel matrix derivative: a meta-analysis. J Periodontal Res.

[17] Harris RJ (2001). Clinical evaluation of 3 techniques to augment keratinized tissue without root coverage. J Periodontol.

[18] Henriques PS, Pelegrine AA, Nogueira AA, Borghi MM (2010). Application of subepithelial connective tissue graft with or without enamel matrix derivative for root coverage: a split-mouth randomized study. J Oral Sci.

[19] Roman A, Soancă A, Kasaj A, Stratul SI (2013). Subepithelial connective tissue graft with or without enamel matrix derivative for the treatment of miller class I and II gingival recessions: a controlled randomized clinical trial. J Periodontal Res.

[20] Rasperini G, Roccuzzo M, Francetti L, Acunzo R, Consonni D, Silvestri M (2011). Subepithelial connective tissue graft for treatment of gingival recessions with and without enamel matrix derivative: a multicenter, randomized controlled clinical trial. Int J Periodontics Restorative Dent.

[21] Agudio G, Cortellini P, Buti J, Pini PG (2016). Periodontal conditions of sites treated with gingival augmentation surgery compared with untreated contralateral homologous sites: an 18- to 35-year long-term study. J Periodontol.

[22] Agudio G, Chambrone L, Pini PG (2017). Biologic remodeling of periodontal dimensions of areas treated with gingival augmentation procedure: a 25-year follow-up observation. J Periodontol.

[23] Pini Prato GP, Franceschi D, Cortellini P, Chambrone L (2018). Long-term evaluation (20 years) of the outcomes of subepithelial connective tissue graft plus coronally advanced flap in the treatment of maxillary single recession-type defects. J Periodontol.

[24] Zucchelli G, De Sanctis M (2005). Long-term outcome following treatment of multiple miller class I and II recession defects in esthetic areas of the mouth. J Periodontol.

